# Prognostic Value, Immune Signature, and Molecular Mechanisms of the PHLDA Family in Pancreatic Adenocarcinoma

**DOI:** 10.3390/ijms231810316

**Published:** 2022-09-07

**Authors:** Yunjie Duan, Yongxing Du, Zongting Gu, Xiaohao Zheng, Chengfeng Wang

**Affiliations:** 1 State Key Lab of Molecular Oncology and Department of Pancreatic and Gastric Surgery, National Cancer Center/National Clinical Research Center for Cancer/Cancer Hospital, Chinese Academy of Medical Sciences and Peking Union Medical College, 17 Panjiayuan Nanli, Chaoyang District, Beijing 100021, China; 2 Department of Hepatobiliary and Pancreatic Surgery and Minimally Invasive Surgery, Zhejiang Provincial People’s Hospital, Hangzhou Medical College, Hangzhou 310000, China

**Keywords:** pancreatic adenocarcinoma, PHLDA family, TP53, biomarkers, prognosis, immune infiltration

## Abstract

Background: Increasing evidence supports the belief that the pleckstrin homology domain family A (PHLDA) family is associated with the development of a variety of cancers. However, the function of the PHLDA family members in PAAD is still unclear. Methods: Comprehensive bioinformatic analyses using R (version 3.6.3), Cytoscape (version 3.9.1), UALCAN, etc., were performed to study the clinicopathological characteristics, prognostic value, immune features, and functional mechanisms of the PHLDA family members in PAAD. Results: The PHLDA family members showed significantly elevated expression in PAAD compared with paracancerous or normal tissues. Their high expression or amplification were significantly correlated with worse clinicopathological characteristics and prognosis in PAAD patients. In addition, the role of the PHLDA family members in the immune regulation is diverse and complex. Mechanistically, TP53 mutations were significantly associated with the promoter methylation and expression levels of the PHLDA family members, which were activated in multiple oncogenic pathways, including the EMT, RAS/MAPK, and TSC/mTOR pathways. Moreover, we found that their expression levels were significantly correlated with the sensitivity of multiple traditional chemotherapeutic drugs and novel targeted MEK1/2 inhibitors. Conclusion: The PHLDA family members play an oncogenic role in the development of PAAD and might serve as new biomarkers or therapeutic targets.

## 1. Introduction

PAAD is a fatal digestive malignancy with a 5-year overall survival rate of approximately 7% [1,2]. Among cancer-related causes of death, this malignancy ranks fourth in the United States and sixth in China [1,3]. Approximately 80% of patients are diagnosed at a late stage with locally advanced or widespread tumors, thus missing the opportunity for radical resection [1,4]. Despite advances in imaging diagnostic techniques, such as endosonography, computed tomography, magnetic resonance imaging, and endoscopic retrograde cholangiography, the early diagnosis and differential diagnosis of PAAD remain challenging for clinicians [5]. At present, there are no specific tumor markers for the diagnosis of PAAD. Mucin-associated markers such as CA 19-9 are currently the most widely used tumor markers for PAAD, but their value as screening markers is limited by their low specificity [6]. In addition, over the last ten years, despite advances in antimetabolite therapy, targeted therapy, and immunotherapy for PAAD, the overall survival rate of patients has not significantly improved due to the late pathological stage at diagnosis, its highly invasive phenotype, and its resistance to chemotherapy [7]. Therefore, the need to explore new therapeutic targets and prognostic biomarkers to improve the prognosis of PAAD patients is urgent.

The pleckstrin homology-like domain (PHLD) protein is a multifunctional protein containing the PH domain that binds to the phosphorylated head group of phosphatidylinositol 4,5-bisphosphate (PtdI(4,5)P2) lipids with high in vitro specificity [8]. The PHLDA family members express the PHLD protein and consist of three members: PHLDA1, PHLDA2, and PHLDA3 [9]. The PHLDA1 gene was originally discovered in T-cell hybridomas and encodes an evolutionarily conserved protein that is rich in proline and histidine residues [10,11]. It has been shown that the PH domain of the PHLDA1 protein can inhibit Akt function by binding to phosphatidylinositol (PIP), which can inhibit tumor progression [12]. However, a study by Sakthianandeswaren et al. found that the expression of PHLDA1 also contributes to the development of intestinal tumors and is associated with the high metastatic potential of osteosarcoma cells [13,14]. PHLDA2 is the first apoptosis-related gene to be imprinted and expressed exclusively from the maternal allele during normal development [15,16]. PHLDA2 is located in the important tumor suppressor gene region 11p15.5 and is believed to have an anticancer effect in lung cancer [17,18]. However, its function in cancer is complex; for example, Moon HG et al. found that PHLDA2 knockdown could inhibit the invasion and proliferation of breast cancer [19]. PHLDA3 is a p53-regulated Akt repressor that competes with the PH domain of Akt for binding to membrane lipids, thereby inhibiting Akt translocation to the cell membrane and activation [20].

However, the study of Lei et al. found that PHLDA3 could also promote the proliferation and invasion of lung adenocarcinoma cells by activating the Wnt signaling pathway [21]. Taken together, the above studies indicate that the PHLDA family members may have different functions in different tumor tissues and cell types, and their specific effects depend on the tumor cells and environment [9]. Most importantly, the role of the PHLDA family members in PAAD is still unclear, and relevant studies are very scarce. This emphasizes the importance of carrying out bioinformatics analyses of the function of the PHLDA family in PAAD.

In the present study, we comprehensively investigated the possible functions and mechanisms of PHLDA family members in the development and progression of PAAD using public databases and multiple bioinformatics analysis techniques. First, we used The Cancer Genome Atlas (TCGA) and Genotype-Tissue Expression (GTEx) databases to analyze the differences in the expression of the PHLDA family members in PAAD samples compared with paracancerous samples and normal pancreas samples. We then utilized the HPA database to explore the protein levels of the PHLDA family members in PAAD tissues. In addition, we analyzed the relationship between their expression level and clinicopathological features, overall survival, and drug sensitivity in patients with PAAD based on the UALCAN database and Kaplan-Meier Plotter database. Potential mechanisms of involvement of PHLDA family members in the development and progression of PAAD were explored by gene variant, immune infiltration, gene enrichment, PPI, and promoter methylation analyses. In summary, our study revealed that the PHLDA family members play an oncogenic role in the occurrence and development of PAAD and are expected to become novel biomarkers and therapeutic targets for patients with this dismal malignancy.

## 2. Results

### 2.1. Aberrant Expression of the PHLDA Family Members across Cancers

**Figure 1:**
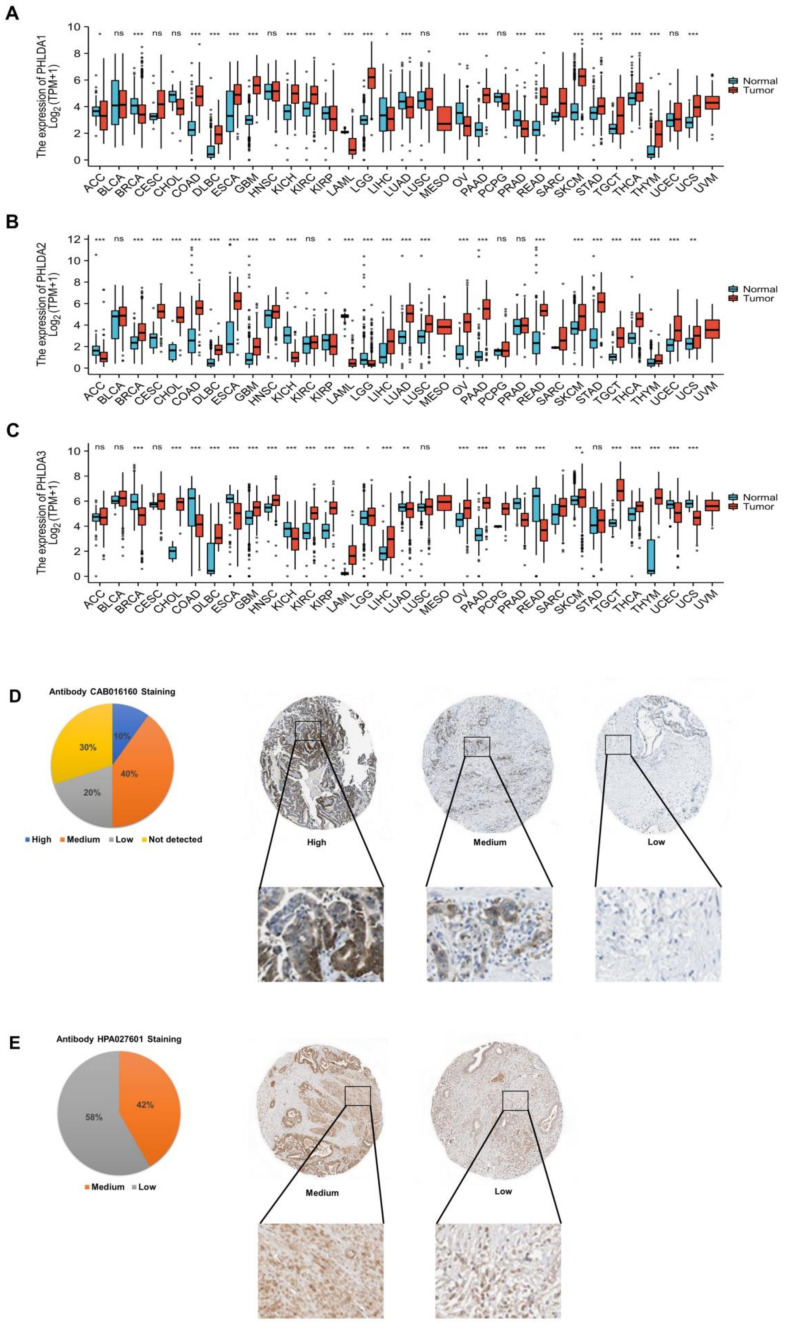
Expression analysis of the PHLDA family in pan-cancer and HPA analysis of PHLDA1/3 in PAAD. (**A**) The expression level of PHLDA1; (**B**) the expression level of PHLDA2; (**C**) the expression level of PHLDA3. ns, *p* ≥ 0.05; * *p* < 0.05; ** *p* < 0.01; *** *p* < 0.001. (**D**) The protein level of PHLDA1 (40×); (**E**) the protein level of PHLDA3 (40×).

To investigate the transcript levels of the PHLDA family members in a variety of cancerous tissues, adjacent paracancerous tissues, and normal tissues, we performed an analysis based on the data from the TCGA database and the GTEx database. The results showed that compared with paracancerous tissues and normal tissues, the transcription levels of the PHLDA family members were significantly increased in 29 types of cancer tissue, including PAAD, glioblastoma multiforme (GBM), and thyroid carcinoma (THCA), but were significantly decreased in 15 types of cancer tissue, including adrenocortical carcinoma (ACC), breast invasive carcinoma (BRCA), and colon adenocarcinoma (COAD) (Figure 1A,B,C). The above findings indicate that the expression of the PHLDA family members was different across cancers and may play diverse roles in different cancers.

### 2.2. Abnormal Expression of PHLDA1/3 Proteins in PAAD Tissues

To investigate the expression of the PHLDA family proteins in PAAD tissues, we used the data of the PAAD project from the HPA database to analyze the immunohistochemical staining of PHLDA1/3 proteins (the PHLDA2 data were missing). The cancer tissues of 10 PAAD patients were stained with the CAB016160 antibody, and the results showed that the PHLDA1 indicator was positive in 7 of the 10 tissue samples. The ratio of high staining–moderate staining–low staining–unstained was 1:4:2:3 (Figure 1D). In addition, the cancer tissues of 12 PAAD patients were stained with the HPA027601 antibody, and the results showed that the PHLDA3 indicators were positive in all of the 12 tissue samples. The ratio of moderate staining to low staining was 5:7 (Figure 1E). Based on the above results, we found that the PHLDA1 protein was positively stained in 70% of the samples, mainly with moderate and high staining, while the PHLDA3 protein was positively stained in all the samples. Thus, PHLDA1/3 proteins were both highly expressed in PAAD tissues, which was consistent with their expression at the transcriptional level.

### 2.3. The Relationship between PHLDA Family Member Expression and the Clinicopathological Characteristics and Prognosis of PAAD Patients

**Figure 2:**
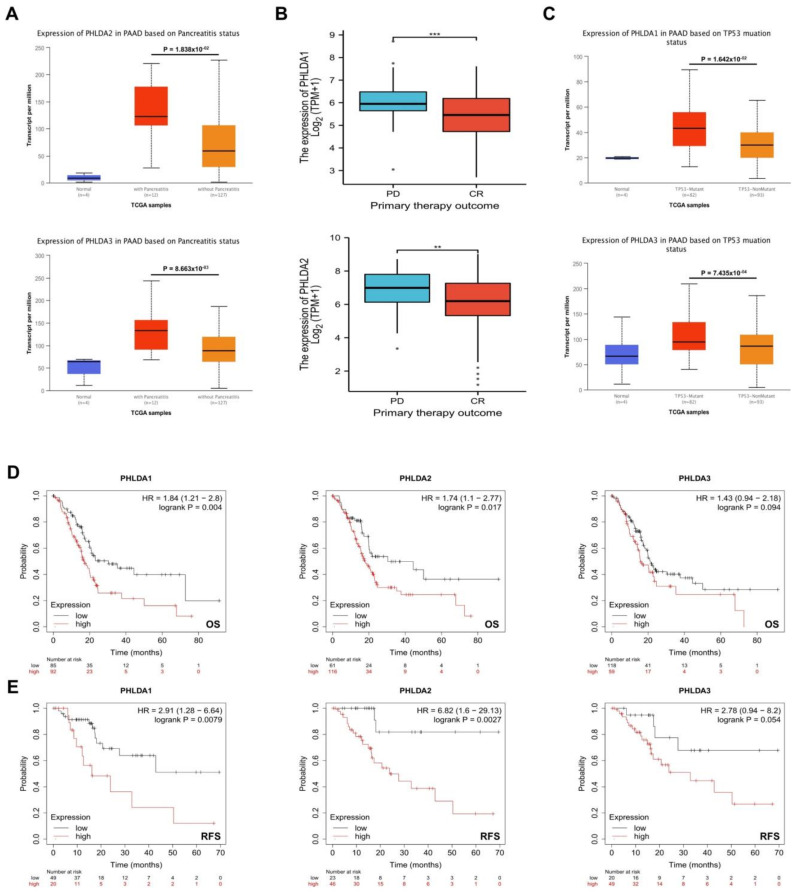
The relationship between expression levels of PHLDA family members and clinicopathologic features, TP53 mutation status, and prognosis of PAAD patients. (**A**) High expression of PHLDA2/3 was significantly correlated with a previous history of pancreatitis; (**B**) high expression of PHLDA1/2 was significantly correlated with a worse initial treatment effect; (**C**) the expression levels of PHLDA1/3 in the TP53 mutant PAAD tissues were significantly higher than those in the TP53 wild−type PAAD tissues; (**D**,**E**) the increased expression levels of PHLDA1 and PHLDA2 were significantly correlated with shorter OS and RFS. ** *p* < 0.01; *** *p* < 0.001.

First, we analyzed the relationship between the expression of PHLDA family members and the clinicopathological characteristics of patients with PAAD using R (version 3.6.3) based on the PAAD project data in the UALCAN database. Our results showed that high PHLDA 2/3 expression was significantly associated with a history of pancreatitis (*p* = 1.838 × 10^−2^/*p* = 8.663 × 10^−3^) (Figure 2A), and the high expression of PHLDA1/2 was significantly correlated with a worse initial treatment effect (*p* = 0.003/*p* = 0.045) (Figure 2B). In addition, it is very interesting that PHLDA 1/3 is expressed at significantly higher levels in TP53 mutant PAAD tissues than in TP53 wild-type tumors (*p* = 1.642 × 10^−2^/*p* = 7.435 × 10^−4^) (Figure 2C). However, the expression levels of the PHLDA family members were not significantly correlated with any of these clinicopathological features, such as tumor grade, tumor stage, N stage, or diabetes history (Supplementary Figure S1).

Next, we analyzed the correlation of the expression of PHLDA family members and the prognosis of patients with PAAD using the Kaplan-Meier Plotter database. The results showed that the increased expression levels of PHLDA1 and PHLDA2 were significantly correlated with shorter OS and RFS (*p* < 0.05) (Figure 2D,E). In conclusion, the present study found that high expression of the PHLDA family members in PAAD patients was related to poor clinicopathological features and prognosis. This suggests that the PHLDA family members might play oncogenic roles and could serve as novel biomarkers in PAAD.

### 2.4. Mutations in the PHLDA Family Member Genes Were Associated with Worse Clinicopathological Characteristics and a Worse Prognosis for PAAD Patients

**Figure 3:**
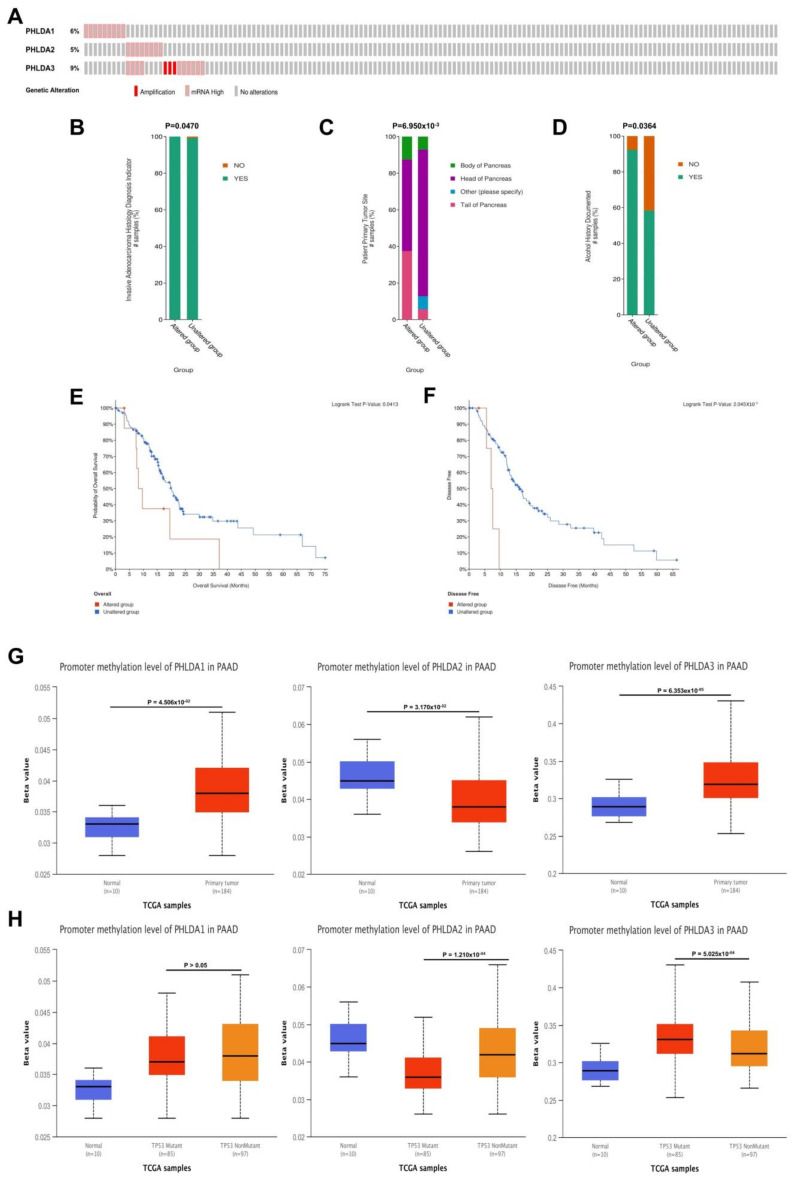
Genetic variation analysis and promoter methylation analysis of the PHLDA family in PAAD. (**A**) Gene variation characteristics of the PHLDA family in PAAD; (**B**,**C**) the mRNA high mutation of PHLDA2 was significantly correlated with PAAD invasion of surrounding tissues and tumor sites; (**D**) the amplification variation of PHLDA3 was significantly correlated with previous drinking history of PAAD patients; (**E**,**F**) PAAD patients with PHLDA1 mRNA high mutation had shorter OS and DFS; (**G**) the promoter methylation levels of PHLDA1/3 in the tumor tissues of patients were significantly higher than those in the paracancerous tissues; (**H**) the promoter methylation level of PHLDA2 was significantly lower in TP53 mutant PAAD tissues than that in TP53 wild-type PAAD tissues, but PHLDA3 was the opposite.

To further explore the related mechanisms by which PHLDA family members are differentially expressed in PAAD, we analyzed gene variants of PHLDA family members using the cBioPortal online tool. Genetic mutations occurred in the PHLDA family member genes in 26 samples (17%) from PAAD patients. Among them, the gene with the highest mutation frequency was PHLDA3 (9%), and the main types of mutations were amplification and mRNA high (Figure 3A). On this basis, we analyzed the clinicopathological characteristics and prognosis of PAAD patients with and without mutations in the PHLDA family member genes. The results showed that there were significant correlations between a high frequency of PHLDA2 mRNA mutations and two clinicopathological features of PAAD: the invasion of surrounding tissues and tumor sites (*p* < 0.05) (Figure 3B,C). Moreover, there was a significant correlation between the amplification mutations of PHLDA3 and a history of drinking in PAAD patients (*p* < 0.05) (Figure 3D). In terms of prognosis, PAAD patients with a high frequency of PHLDA1 mRNA mutations had shorter OS and DFS (*p* < 0.05) (Figure 3E,F), but PHLDA2/3 amplification mutations did not significantly affect the OS and DFS of PAAD patients (Supplementary Figure S2).

### 2.5. Correlations between the Methylation Levels of the PHLDA Family Member Promoters and the Clinicopathological Characteristics of PAAD Patients

We next explored the association of epigenetic variations in PHLDA family members with clinicopathological features of patients with PAAD. Based on the data of the PAAD project from the UALCAN database, we analyzed the relationship between the methylation levels of the PHLDA family member promoters and the clinicopathological characteristics of PAAD patients. As shown in Figure 3G, the promoter methylation levels of PHLDA1/3 in the tumor tissues of patients were significantly higher than those in the paracancerous tissues (*p* = 4.506 × 10^−2^/*p* = 6.353 × 10^−5^). However, the methylation level of the PHLDA2 promoter in paracancerous tissues was significantly higher than that in tumor tissues (*p* = 3.170 × 10^−2^). In addition, we found that the methylation level of the PHLDA2 promoter was significantly decreased in patients with TP53 mutations (*p* = 1.210 × 10^−4^) (Figure 3H), while the methylation level of the PHLDA3 promoter was significantly increased (*p* = 5.025 × 10^−4^) (Figure 3H). Therefore, the effect of TP53 mutations on the epigenetic modification of PHLDA2/3 promoter methylation may be an important factor for their diverse functions in the occurrence and development of PAAD. However, the methylation levels of the PHLDA family member promoters were not associated with the clinicopathological characteristics of tumor stage, tumor grade, or N stage (Supplementary Figure S3).

### 2.6. The Immune Landscape of PHLDA Family Member Expression and Variations in PAAD Patients

**Figure 4:**
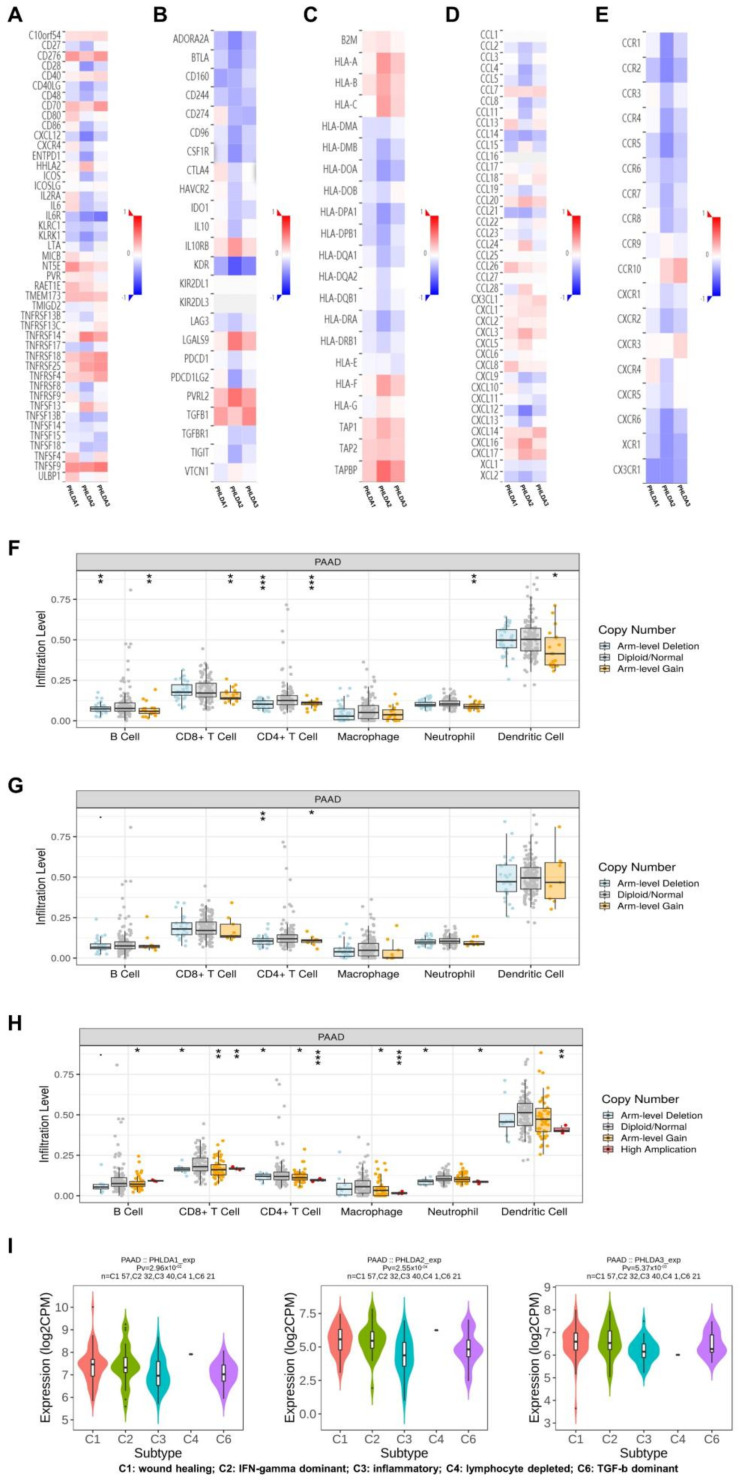
Analysis of the immune infiltration of the PHLDA family in PAAD. (**A**–**E**) PHLDA1 was positively correlated with 23 types of immune promoters, including IL6, NT5E, and TNFSF9, while PHLDA2 was negatively correlated with most immunomodulators (immune promoters, MHC molecules, chemokines, and chemokine receptors); (**F**–**H**) the copy number alteration of PHLDA1/3 may affect the infiltration levels of six types of dominant immune cells; (**I**) expression of PHLDA family was significantly different among the five immune subtypes. ns, *p* ≥ 0.05; * *p* < 0.05; ** *p* < 0.01; *** *p* < 0.001.

Next, using the data of the PAAD project from the TISIDB database, we explored the relationship between the expression of the PHLDA family members and the level of tumor-infiltrating cells and multiple immunomodulators based on various immunological markers in PAAD (Supplementary Material S1). Our results showed that the role of the PHLDA family members in tumor immune regulation was not consistent. First, PHLDA1 was positively correlated with the infiltration levels of a variety of immunocompetent tumor-infiltrating cells, including Act CD4, CD56dim, and Act DC (Table 1, Supplementary Figure S4A), and 23 types of immune promoters, including IL6, NT5E, and TNFSF9 (Figure 4A). Therefore, high expression levels of PHLDA1 may mainly enhance the immune response to PAAD. In contrast, PHLDA2 was negatively correlated with the infiltration levels of several immunocompetent tumor-infiltrating cells, including TemCD8, Th1, and NKT cells (Table 1, Supplementary Figure S4B), and PHLDA2 was negatively correlated with most immunomodulators (immune promoters, MHC molecules, chemokines, and chemokine receptors) in PAAD (Figure 4B–E). The above findings indicate that PHLDA2 mainly plays an immunosuppressive role in the PAAD tumor microenvironment, which might be associated with the immune escape of PAAD. However, PHLDA3 was only associated with the infiltration level of a small number of tumor-infiltrating cells (Table 1, Supplementary Figure S4C), indicating its limited immunomodulatory role in PAAD. In addition, we analyzed published single-cell sequencing datasets for pancreatic cancer (GSE148073 and GSE154778) and the final results show that the expression level of the PHLDA family is high in tumor tissues such as the TFF1 ductal cell and MET epithelial cell, but very low in immune infiltrating cells (Supplementary Figure S5). We speculate that the high expression of the PHLDA family on pancreatic cancer tumor cells may recruit relevant immune cells.

Then, we further explored the relationship between the copy number alterations of the PHLDA family members and the infiltration level of tumor-infiltrating cells based on the data from the PAAD project in the TIMER database. The results showed that copy number alterations of PHLDA1/3 may affect the infiltration level of six dominant immune cells, especially arm-level gain and high amplification (Figure 4F,G,H) (Supplementary Material S2).

Finally, we investigated the relationship between the expression levels of the PHLDA family members and immune subtypes. As shown in Figure 4I, the expression levels of the PHLDA family members were significantly different among the five types of immune subtypes. Among them, the highest expression level of the PHLDA family members was found in the PAAD tissues of the C1 subtype, while the lowest expression level of the PHLDA family members was found in the PAAD tissues of the C3 subtype.

In summary, the role of the PHLDA family members in the immune regulation of the PAAD tumor microenvironment is complex and diverse. It is necessary to pay special attention to the immune escape of PAAD cells caused by the high expression of PHLDA2, which may be a potential target for the treatment of PAAD.

### 2.7. Enrichment Analysis of the PHLDA Family Members and the 600 Co-Expressed Genes

**Figure 5:**
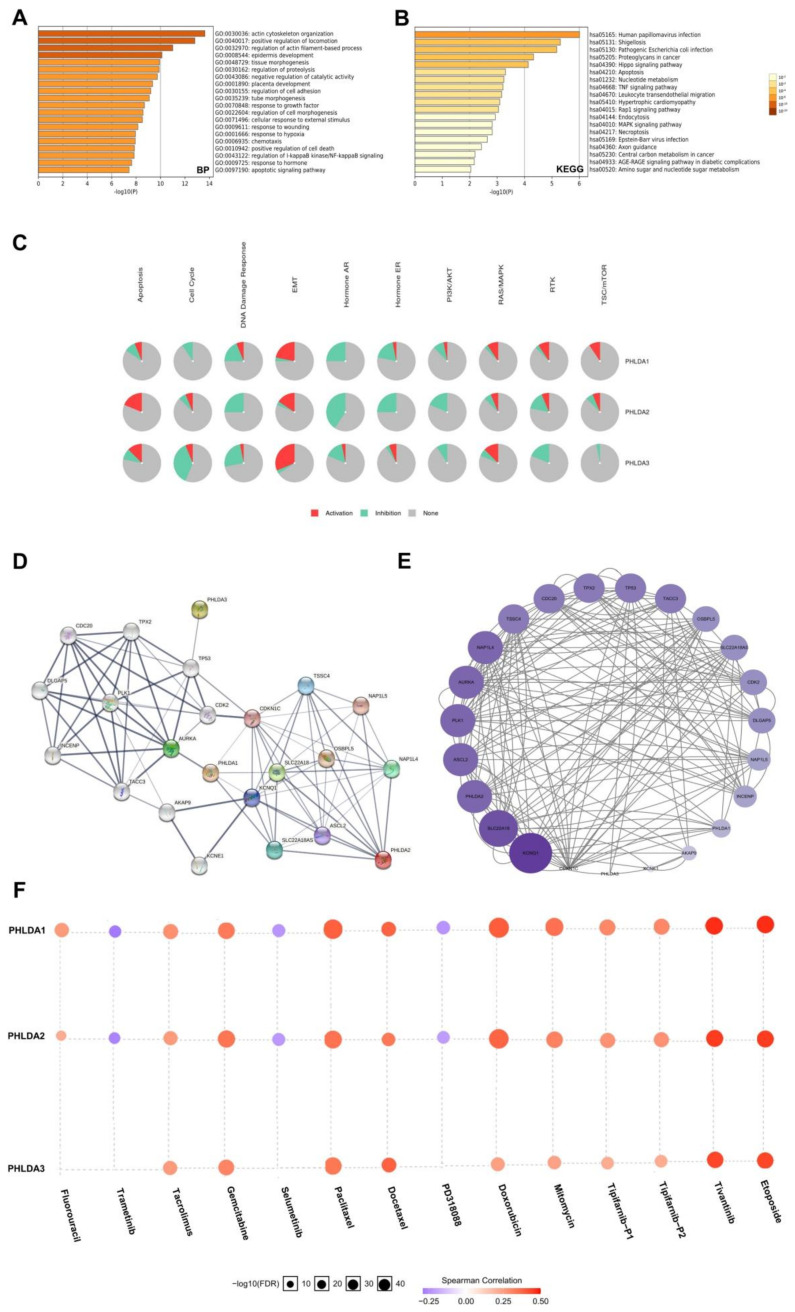
Gene enrichment analysis, PPI network construction, and drug sensitivity analysis of PHLDA Family in PAAD. (**A**) The GO enrichment of the BP terms of the PHLDA family and its 600 co-expressed genes; (**B**) the KEGG enrichment of the PHLDA family and its 600 co-expressed genes; (**C**) the PHLDA family played an activating role in a variety of oncogenic pathways; (**D**,**E**) TP53 played an important role in PPI network, which was closely related to the PHLDA family; (**F**) the expression of PHLDA family was negatively correlated with the sensitivity of various PAAD-targeting or chemotherapeutic drugs, including gemcitabine, docetaxel, and fluorouracil.

To further explore the functional mechanism of the PHLDA family members in the development of PAAD, we performed GO and KEGG enrichment analyses (Supplementary Material S4) on the PHLDA family members and their 600 co-expressed genes (Supplementary Material S3), which were based on the data from the PAAD project in the LinkedOmics database and Metascape database. GO analysis showed that the PHLDA family members and their 600 co-expressed genes were mainly enriched in the BP terms “actin cytoskeleton organization”, “positive regulation of locomotion”, and “regulation of actin filament-based process” (Figure 5A), the CC terms “cell-substrate junction”, “cell-cell junction”, and “actin cytoskeleton” (Supplementary Figure S6A), and the MF terms “cadherin binding”, “protein domain specific binding”, and “kinase binding” (Supplementary Figure S6B). KEGG analysis showed that the PHLDA family members and their 600 co-expressed genes may play important roles in various pathways related to the occurrence and development of cancer, such as the “Hippo signaling pathway”, “apoptosis”, the “TNF signaling pathway”, and the “MAPK signaling pathway” (Figure 5B). On this basis, we used the data of the PAAD project from the GSCA database to further analyze the mechanism of action of the PHLDA family members in a variety of oncogenic pathways. As shown in Figure 5C, the PHLDA family played an active role in the EMT signaling pathway, and PHLDA1 was an important activator of the “RAS/MAPK signaling pathway”, “RTK signaling pathway”, and “TSC/mTOR signaling pathway”. In addition, the PHLDA family was mainly involved in the “DNA damage response signaling pathway”, “hormone AR signaling pathway”, and “PI3K/AKT signaling pathway”. The above findings reveal a possible mechanism by which the PHLDA family members may promote the development of pancreatic cancer, namely, by activating multiple oncogenic pathways and enhancing the survival and motility of PAAD cells.

### 2.8. Construction and Analysis of the PPI Network Associated with the PHLDA Family Members

To construct and analyze the PPI network of the PHLDA family members in PAAD patients, we used the STRING database to identify the 23 types of genes that have the strongest PPIs with the PHLDA family members (Figure 5D) (Supplementary Material S5) and then mapped the related PPI network using Cytoscape software. The larger circles and darker colors indicate that the number of PPIs associated with the gene was higher. The results showed that TP53 demonstrated the higher score in the PPI network of the PHLDA family members (Figure 5E) (Supplementary Material S6), suggesting that TP53 plays an important role in the PPI network closely related to the PHLDA family members. In addition, an interesting finding was that there was a direct PPI interaction between PHLDA3 and TP53 (Figure 5D), which suggests that PHLDA3 may be involved in the biological function of TP53 in PAAD.

### 2.9. The Expression Levels of the PHLDA Family Members Affect the Treatment Sensitivity of Multiple Drugs

Finally, we analyzed the associations between the expression levels of the PHLDA family members and the therapeutic sensitivity to various chemotherapeutic and targeted drugs using the GSCALite database. The results are shown in Figure 5F, and the expression levels of the PHLDA family members were negatively correlated with the sensitivity to various targeted or chemotherapeutic drugs for PAAD, including gemcitabine, docetaxel, and fluorouracil. Therefore, there is a good prospect for the PHLDA family members to serve as a therapeutic target for PAAD in drug development. In addition, an interesting finding was that the expression levels of PHLDA1/2 were positively correlated with the sensitivity to three targeted drugs, namely, selumetinib, trametinib, and PD318088, all of which are MEK1/2 inhibitors. We searched the ClinicalTrials.gov database (https://clinicaltrials.gov/) (accessed on 1 July 2022) for clinical studies related to the above three drugs and found that clinical trials of selumetinib and trametinib in the treatment of PAAD are in progress, but the clinical trial of PD318088 in the treatment of PAAD has not yet been carried out. As a promising biomarker for the prediction of the sensitivity to multiple drugs for PAAD, further experimental studies on the PHLDA family members should be carried out as soon as possible. The results of these studies are highly anticipated.

## 3. Discussion

In recent years, people have paid increasing attention to the function of the PHLDA family members in tumors. Relevant studies have found that PHLDA family members have potential tumor suppressor or cancer-promoting roles in various malignancies, such as breast, rectal, and lung cancer [13,21,22]. However, functional studies of PHLDA family members in PAAD are currently lacking, and no specific bioinformatics analysis has been performed. This study is the first bioinformatics analysis of the function of the PHLDA family members in PAAD and comprehensively reveals the functions and possible mechanisms of PHLDA family members involved in the development and progression of PAAD from the aspects of gene expression, gene variation, promoter methylation, immune cell infiltration, gene function, pathway enrichment, PPI and drug sensitivity.

As members of the PHLD protein family, the expression products of the PHLDA family members all contain the PH domain [9]. This domain can compete with the PH domain of Akt to bind membrane lipids to inhibit the activation of the Akt signaling pathway, thus playing an inhibitory role in a variety of tumors [9]. However, related studies have found that the PH domain can also promote the proliferation and migration of tumor cells [23]. Therefore, it is necessary to explore the function and molecular mechanism of the PH domain in PAAD. Interestingly, our study found that members of the PHLDA family can activate the EMT signaling pathway. The EMT signaling pathway is a classic cancer-promoting pathway that leads to the formation of secondary metastatic lesions by activating the movement and invasive ability of tumor cells in many tumors, including pancreatic cancer, prostate cancer, and breast cancer [24,25,26,27]. Therefore, we speculate that the PH domain of the PHLDA family expression products may be an important factor in activating the EMT signaling pathway, and this activation may be the potential mechanism by which the PHLDA family acts in promoting the occurrence and development of PAAD, which needs further experimental verification. In addition, PHLDA1 mainly played a stimulating role in the RAS/MAPK, RTK, and TSC/mTOR signaling pathways. The RAS/MAPK and RTK signaling pathways can promote the growth and proliferation of tumor cells [28,29], and the TSC/mTOR signaling pathway can promote tumor angiogenesis [30]. In short, the PHLDA family members may promote the occurrence and development of PAAD by activating a variety of carcinogenic pathways.

Next, it is worth noting that we discovered a possible target for the occurrence and development of PAAD driven by TP53 mutations. TP53, located on the short arm of human chromosome 17, is a tumor suppressor gene and the most common mutant gene in the human cancer cell [31]. In addition, TP53 mutations can be detected in 50–60% of human cancers, and some mutated proteins play a cancer-promoting role in tumors such as colon cancer, head and neck squamous cell carcinoma, and lung adenocarcinoma [32,33,34]. Thus, it is an important prognostic and predictive marker in cancer. In this study, we found that TP53 plays an important role in the PPI network of the PHLDA family members, and its expression products interact with a variety of proteins. Based on this rich interaction, the functional changes in proteins caused by TP53 mutations will inevitably affect the function of many interacting proteins and will further lead to the functional disorder of many interaction pathways [31]. Interestingly, this study also found that TP53 mutations were significantly correlated with the increased expression of PHLDA1 and PHLDA3 in PAAD, and the high expression of PHLDA1/3 was associated with poor prognosis in patients with PAAD. These findings suggest that PHLDA1/3 may be potential downstream targets of mutant TP53 that promote the occurrence and development of PAAD. This finding is helpful for researchers to further understand the mechanism by which TP53 mutations cause PAAD. Further study of this potential mechanism is expected to find a new target that can prevent the occurrence and development of PAAD driven by TP53 mutations, thus improving the prognosis of patients. Another concern is that we found that TP53 mutations may affect the promoter methylation levels of most members of the PHLDA family, and whether this effect can promote the occurrence and development of PAAD is worthy of future in-depth study. In addition, it is traditionally believed that CpG methylation in the promoter region is usually related to the inactivation of gene expression, so it is generally regarded as an inhibitory marker. However, recent studies have found that the function of DNA methylation in varied tissues and different gene regions is diverse [35,36,37]. Our study found that compared with TP53 wild-type PAAD tissues, the promoter methylation level and expression level of PHLDA3 in TP53 mutant PAAD tissues were both increased. These findings indicate that the methylation of the PHLDA3 promoter might increase its expression in PAAD.

Finally, we analyzed the role of the PHLDA family in the immunity of pancreatic cancer, and explored the relationship between the expression level of the PHLDA family and pancreatic cancer targeting or chemotherapeutic drug sensitivity. The final analysis results show that the PHLDA family is closely related to the clinical treatment of pancreatic cancer. Firstly, the immune system is complex. In addition to acting as a first line of defense against various pathogens, immune cells can provide surveillance functions by recognizing and destroying latent cancer cells. Immunocompetent tumor-infiltrating cells are a unique type of immune cells [38] that infiltrate the tumor microenvironment by detecting tumor antigens and releasing proinflammatory and immune molecules that regulate immune function. In addition, although cancer immunotherapy has been shown to improve survival in a variety of cancers, remission rates in patients with PAAD remain low [39]. Therefore, a comprehensive study of the interaction between tumors and immune cells will help to clarify the pathogenesis of cancer and develop a new immunotherapy strategy. We studied the relationship between the infiltration levels of immunocompetent tumor-infiltrating cells and the expression levels of the PHLDA family members in PAAD. The results show that the roles of PHLDA1 and PHLDA2 in PAAD immune regulation were opposite, which indicated that the roles of the PHLDA family members in PAAD tumor immune regulation are diverse and complex. Similarly, Bolandi et al. have also found that members of the B7 family may play different roles in tumor immune regulation [40]. This finding indicates that our incidence of members of the same family playing different roles in tumor immune regulation is not an isolated case, which may be very important to maintaining the balance between immune effectiveness and autoimmune suppression [40]. However, regardless of whether their regulatory functions are consistent, the functions of PHLDA1/2 in the tumor immunoregulation of PAAD are very active. For example, PHLDA1 was positively correlated with the infiltration levels of a variety of immunocompetent tumor-infiltrating cells, including Act CD4, CD56dim, and Act DC and 23 types of immune promoters, including IL6, NT5E, and TNFSF9. Therefore, high expression levels of PHLDA1 may mainly enhance the immune response to PAAD. In contrast, PHLDA2 was negatively correlated with the infiltration levels of several immunocompetent tumor-infiltrating cells, including TemCD8, Th1, and NKT cells, and PHLDA2 was negatively correlated with most immunomodulators (immune promoters, MHC molecules, chemokines, and chemokine receptors) in PAAD, so they can be used as potential targets of PAAD immunotherapy and molecular indicators to predict the efficacy of immunotherapy. In addition, our study revealed that the expression of the PHLDA family members was negatively correlated with the sensitivity to a variety of PAAD-targeting or chemotherapeutic drugs, including gemcitabine, docetaxel, and fluorouracil. Therefore, the research and development of PAAD-targeting drugs for the PHLDA family members will have good prospects. In addition, an interesting finding was that the expression levels of PHLDA1/2 were positively correlated with the sensitivities to three MEK 1/2 inhibitors, including selumetinib, trametinib, and PD318088. Previous studies have found that the activation of the MEK/ERK/P21 signaling pathway can promote the development of PAAD [41], and MEK inhibitors can activate the immune recognition of PAAD to inhibit the progression of PAAD [42]. In addition, clinical trials have confirmed that trametinib inhibits PAAD tumors [43]. Therefore, for PAAD patients with high expression of PHLDA1/2, it may be better to choose MEK1/2 inhibitors for treatment.

This research is the first bioinformatics analysis of the functions of the PHLDA family members in PAAD. We found obvious differences in the expression levels of the PHLDA family members across cancers, and these proteins may play diverse roles in different cancers. The high levels of expression and amplification mutations of the PHLDA family members are significantly related to worse clinicopathological features and a worse prognosis for PAAD patients. In addition, the PHLDA family members may promote the occurrence and development of PAAD by activating a variety of oncogenic pathways and regulating PAAD tumor immunity. Another finding is that TP53 mutations have a significant effect on the promoter methylation levels of most members of the PHLDA family, and PHLDA1/3 may be potential downstream targets to drive the occurrence and development of PAAD with TP53 mutations. Moreover, we found that the PHLDA family members are associated with the sensitivity to a variety of PAAD-targeted or chemotherapeutic drug treatments. Therefore, the PHLDA family members could be used as targets for the development of therapeutic drugs for PAAD or as molecular markers for predicting drug sensitivity. However, the present study has certain limitations. For example, the number of databases included in this study is somewhat inadequate. Moreover, the present study is just a bioinformatics analysis of the functions and mechanisms of the PHLDA family involved in the development and progression of PAAD. Future experimental studies could further confirm the tumor-promoting role of PHLDA family members in PAAD.

## 4. Methods and Materials 

Ethics statement: This study was approved by the Ethics Committee of the Cancer Hospital, Chinese Academy of Medical Sciences and was conducted strictly according to the principles of the Declaration of Helsinki. All data in this study were retrieved from online databases. No human or animal tests were involved.

### 4.1. Expression Analysis

In this study, R (version 3.6.3) was used to analyze the expression levels of the PHLDA family member genes in cancerous and paracancerous tissues in the PAAD project of the TCGA database and normal pancreas of the GTEx database (https://commonfund.nih.gov/GTEx) (accessed on 2 July 2022). The statistical significance of the difference in expression was evaluated by the Wilcoxon test. Then, the UALCAN database (http://ualcan.path.uab.edu) (accessed on 4 July 2022) was used to obtain the expression levels of the PHLDA family member genes in PAAD patients with different clinicopathological characteristics, and the statistical analysis was performed by Welch’s *t* test. *p* < 0.05 was considered statistically significant [44,45].

### 4.2. HPA Analysis

The immunohistochemical staining of PHLDA1/2 in PAAD tissues was analyzed using the HPA database (https://www.proteinatlas.org/) (accessed on 5 July 2022). According to dyeing intensity and quantification, the protein expression levels were classified into four categories. The classification criteria for the protein expression levels were as follows: negative, not detected; weak and <25%, not detected; weak combined with either 25–75% or 75%, low; moderate, and <25%, low; moderate combined with either 25–75% or 75%, medium; strong and <25%, medium; and strong combined with either 25–75% or 75%, high [46].

### 4.3. Survival Analysis

The Kaplan-Meier Plotter database (http://www.kmplot.com/) (accessed on 6 July 2022) was used to analyze the correlations between PHLDA family member expression levels and overall survival (OS) and recurrence-free survival (RFS) in PAAD [47]. The “survival” R package (version 2.38) was utilized to calculate log-rank P values, and *p* < 0.05 was considered statistically significant [48].

### 4.4. Gene Variation Analysis

The cBioPortal database (http://www.cbioportal.org/) (accessed on 3 July 2022) was used to analyze the genetic variations of the PHLDA family members in PAAD, and the correlations between the variations and some clinicopathological characteristics were further determined [49]. Statistical significance was assessed by the chi-squared test, and *p* < 0.05 was considered statistically significant. In addition, the log-rank test was used to evaluate the statistical significance between PHLDA family member gene mutations and OS and disease-free survival (DFS) in PAAD patients, and *p* < 0.05 was considered statistically significant [50].

### 4.5. Methylation Analysis

To evaluate the associations between the expression levels of the PHLDA family members and the levels of promoter methylation, promoter methylation levels in PAAD under different conditions were analyzed in the UALCAN database. The statistical significance of the difference was evaluated by Welch’s *t* test, and *p* < 0.05 was considered statistically significant [51,52].

### 4.6. Immune Infiltration Analysis

The correlations between the expression levels of the PHLDA family members and the infiltration levels of infiltrating immune cells and the expression levels of immune molecules in PAAD were analyzed using the TISIDB database (http://cis.hku.hk/TISIDB/) (accessed on 7 July 2022). Statistical significance was assessed by the Spearman test, and *p* < 0.05 was considered statistically significant [53]. The database was then used to analyze the differences in the expression levels of the PHLDA family members in different immune subtypes of PAAD. The statistical significance of the difference in expression levels was evaluated by the Kruskal-Wallis test, and *p* < 0.05 was considered statistically significant [53]. In addition, the TIMER database (http://timer.cistrome.org/) (accessed on 8 July 2022) was used to analyze the correlations between the copy number alterations of the PHLDA family members and the infiltration levels of the six types of immune cells in PAAD. The statistical significance of the difference was evaluated by the Wilcoxon rank-sum test, and *p* < 0.05 was considered statistically significant [54,55].

### 4.7. Gene Enrichment Analysis

The LinkedOmics database (http://www.linkedomics.org/) (accessed on 10 July 2022) was used to select the 600 genes most closely related to the co-expression of the PHLDA family members [56]. The Metascape database (https://metascape.org) (accessed on 14 July 2022) was used to visualize the biological process (BP), cellular components (CC), molecular function (MF), and Kyoto Encyclopedia of Genes and Genomes (KEGG) of the PHLDA family members and their 600 co-expressed genes [57]. In addition, pathway enrichment of the PHLDA family members was performed using the GSCALite database (http://bioinfo.life.hust.edu.cn/web/GSCALite/) (accessed on 16 July 2022) [58].

### 4.8. Construction of the Functional PPI Network

The genes with the strongest PPIs with the PHLDA family members were obtained by using the STRING database (https://string-db.org/) (accessed on 18 July 2022), and the functional PPI network of the PHLDA family members was established [59]. Cytoscape (version 3.9.1) was used to score the effect of different genes in the PPI network [60,61]. 

### 4.9. Drug Sensitivity Analysis

The GSCALite database was used to analyze the correlation between the expression levels of the PHLDA family members and a variety of chemotherapy or targeted therapy drugs for PAAD. The statistical significance of the differences was assessed by the Spearman test. *p* < 0.05 was considered statistically significant [58].

## 5. Conclusions

In summary, this comprehensive study on the functions of the PHLDA family members reveals that they might play an oncogenic role in the development and progression of PAAD and serve as new biomarkers or therapeutic targets for patients with PAAD. Further functional verification and mechanism exploration studies should be promptly carried out.

## Figures and Tables

**Table 1:** Relationship between PHLDA family expression and the abundance of tumor-infiltrating immune cells in PAAD.

Description	PHLDA1		PHLDA2		PHLDA3	
	Purity		Purity		Purity	
	Cor	*p*	Cor	*p*	Cor	*p*
Act CD8	−0.073	0.334	−0.363	***	−0.013	0.858
Tcm CD8	0.146	0.0514	0.008	0.911	0.061	0.415
Tem CD8	0.021	0.776	−0.244	**	−0.028	0.715
Act CD4	0.249	***	−0.042	0.576	−0.087	0.247
Tcm CD4	0.341	***	0.209	**	0.298	***
Tem CD4	−0.106	0.158	−0.546	***	−0.313	***
Tfh	−0.05	0.509	−0.294	***	−0.077	0.305
Tgd	0.073	0.329	−0.141	0.0589	0.135	0.0707
Th1	−0.001	0.993	−0.251	***	−0.024	0.755
Th17	0.027	0.722	0.363	***	−0.021	0.782
Th2	0.238	**	−0.157	*	−0.174	*
Treg	0.036	0.627	−0.304	***	−0.051	0.495
Act B	−0.218	**	−0.363	***	−0.142	0.0587
Imm B	−0.088	0.242	−0.326	***	−0.071	0.345
Mem B	−0.042	0.578	−0.429	***	−0.198	**
NK	0.044	0.557	−0.272	***	−0.041	0.587
CD56bright	0.279	***	0.328	***	0.405	***
CD56dim	0.34	***	0.459	***	0.412	***
MDSC	0.018	0.811	−0.171	*	0.034	0.655
NKT	0.051	0.499	−0.243	**	−0.087	0.248
Act DC	0.199	**	0.136	0.0701	0.178	*
pDC	0.257	***	−0.053	0.481	0.049	0.51
iDC	−0.13	0.084	−0.192	*	−0.194	***
Macrophage	0.006	0.932	−0.251	***	0.029	0.696
Eosinophil	−0.106	0.159	−0.379	***	−0.253	***
Mast	0.018	0.812	−0.391	***	−0.119	0.111
Monocyte	0.074	0.325	0.163	*	0.007	0.925
Neutrophil	0.032	0.668	−0.078	0.299	−0.097	0.196

Notes: * *p* < 0.05; ** *p* < 0.01; *** *p* < 0.001.

## Data Availability

The original contributions presented in the study are included in the article/Supplementary Materials; further inquiries can be directed to the corresponding author.

## Supplementary Materials

The following supporting information can be downloaded at: https://www.mdpi.com/article/10.3390/ijms231810316/s1.

## Abbreviations

PAAD: pancreatic adenocarcinoma; PHLDA: pleckstrin homology domain family A; PPI: protein–protein interaction; PHLD: pleckstrin homology-like domain; PIP: phosphatidylinositol; TCGA: The Cancer Genome Atlas; GTEx: Genotype-Tissue Expression; OS: overall survival; RFS: recurrence-free survival; DFS: disease-free survival; BP: biological process; CC: cellular components; MF: molecular function; KEGG: Kyoto Encyclopedia of Genes and Genomes; GBM: glioblastoma multiforme; THCA: thyroid carcinoma; ACC: adrenocortical carcinoma; BRCA: breast invasive carcinoma; COAD: colon adenocarcinoma; PtdI(4,5)P2: phosphatidylinositol 4,5-bisphosphate.
