# Supplementary figures and images for: Prognostic Value, Immune Signature, and Molecular Mechanisms of the PHLDA Family in Pancreatic Adenocarcinoma
Source: Int J Mol Sci. 2022 Sep 7;23(18):10316. doi: 10.3390/ijms231810316 (PMC9499624; doi:10.3390/ijms231810316)

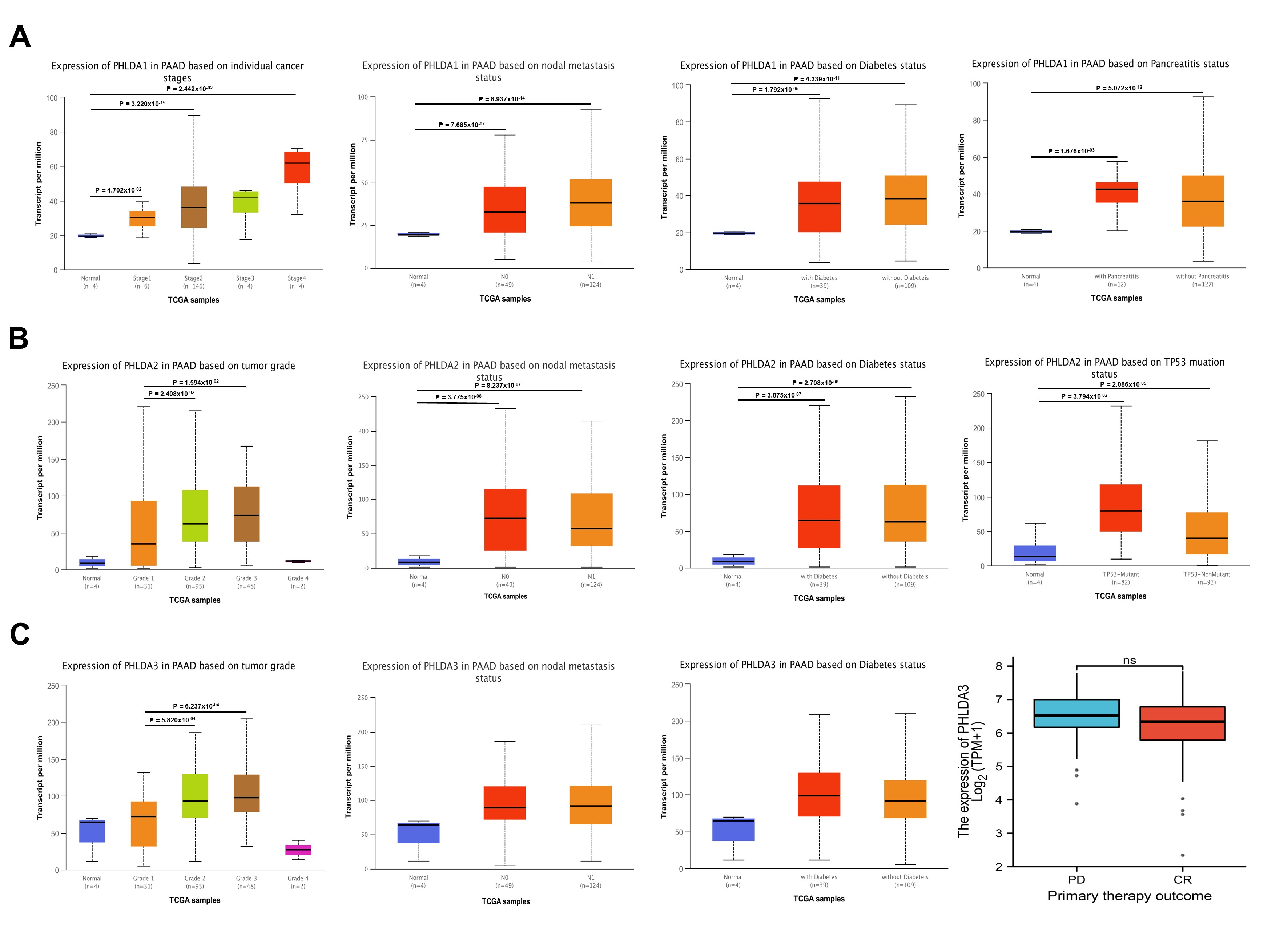

Supplement: Supplementary file 1 [file ijms-23-10316-s001.zip › Supplementary figure S1 The relationship between expression levels of PHLDA family and clinical features.jpg]

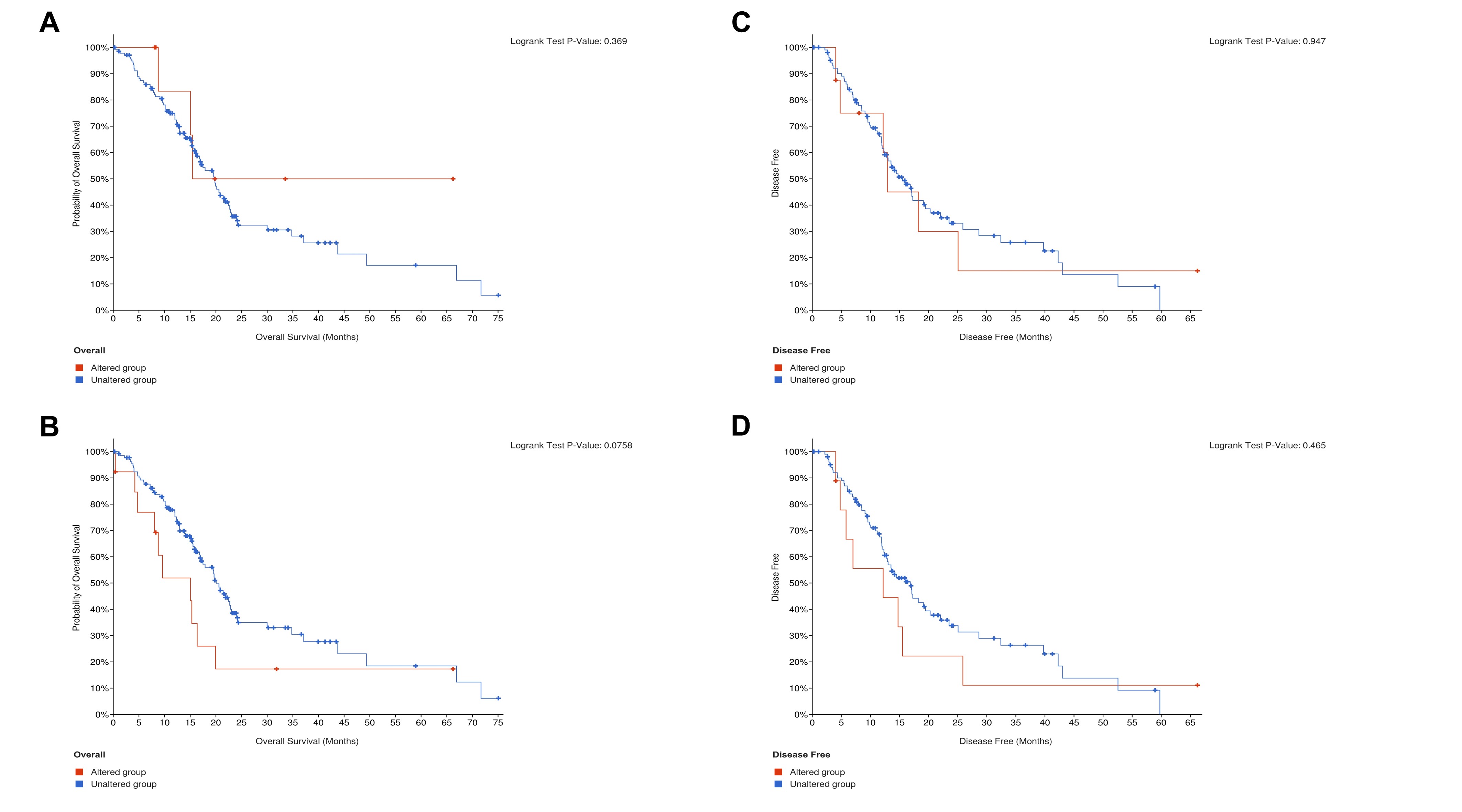

Supplement: Supplementary file 1 [file ijms-23-10316-s001.zip › Supplementary Figure S2 The mRNA high variation or amplification variation of PHLDA2 or 3 had no significant effect on OS and DFS in PAAD patients..jpg]

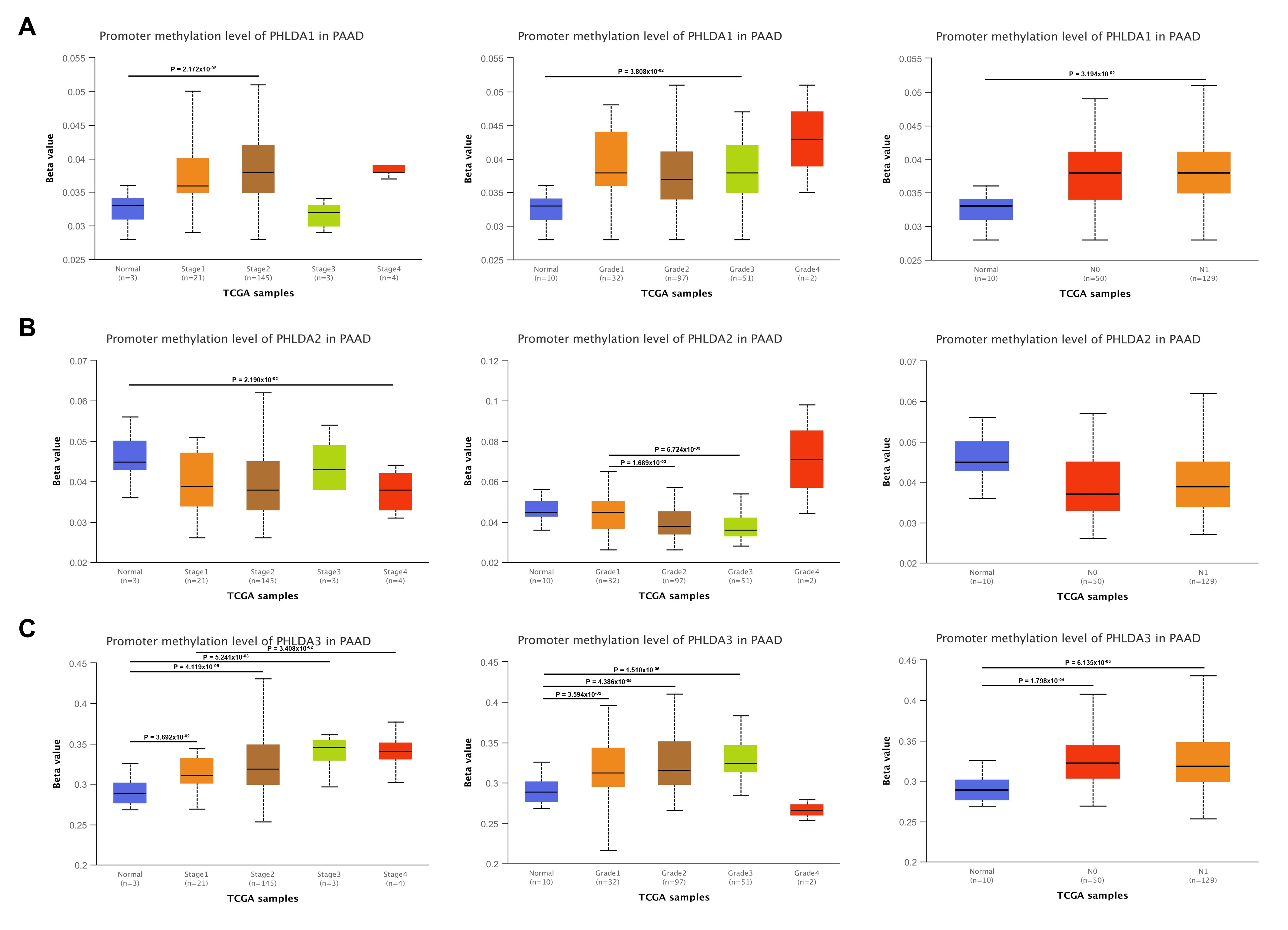

Supplement: Supplementary file 1 [file ijms-23-10316-s001.zip › Supplementary figure S3 The relationship between promoter methylation levels of PHLDA family and clinical features.jpg]

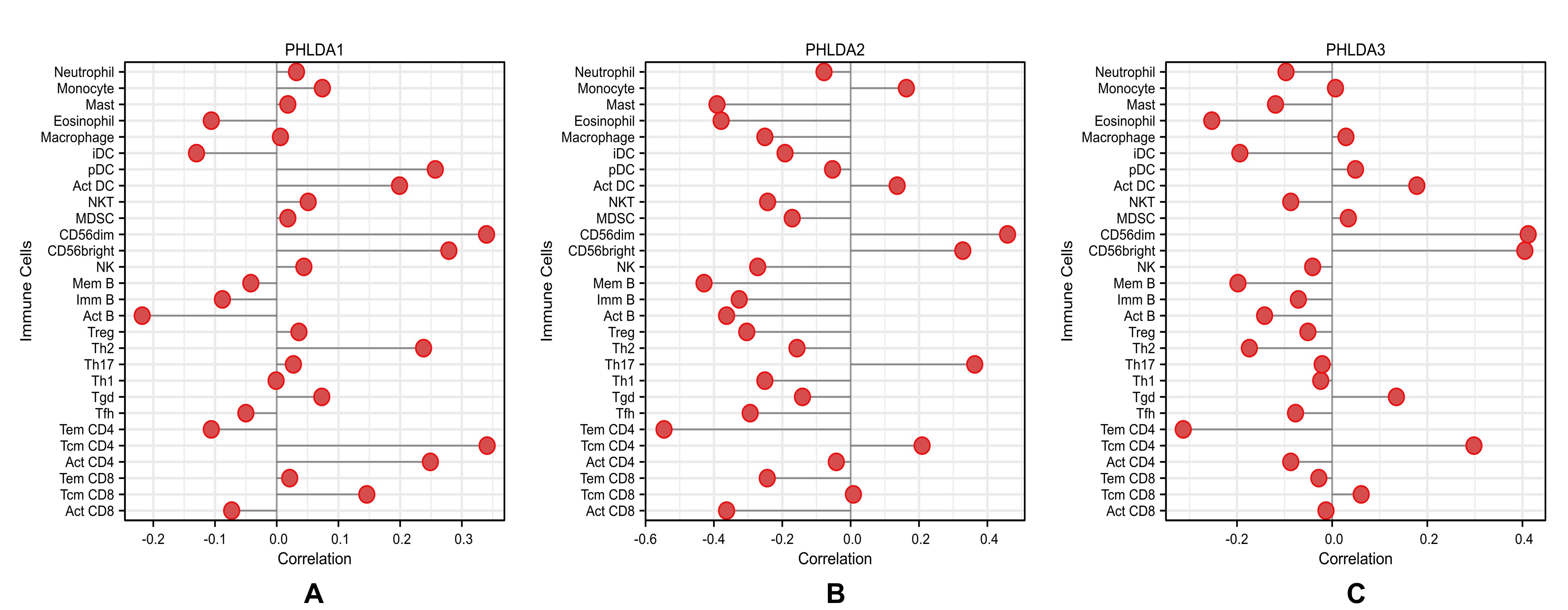

Supplement: Supplementary file 1 [file ijms-23-10316-s001.zip › Supplementary Figure S4 Relationship between PHLDA family expression and the abundance of tumor-infiltrating immune cells in PAAD..jpg]

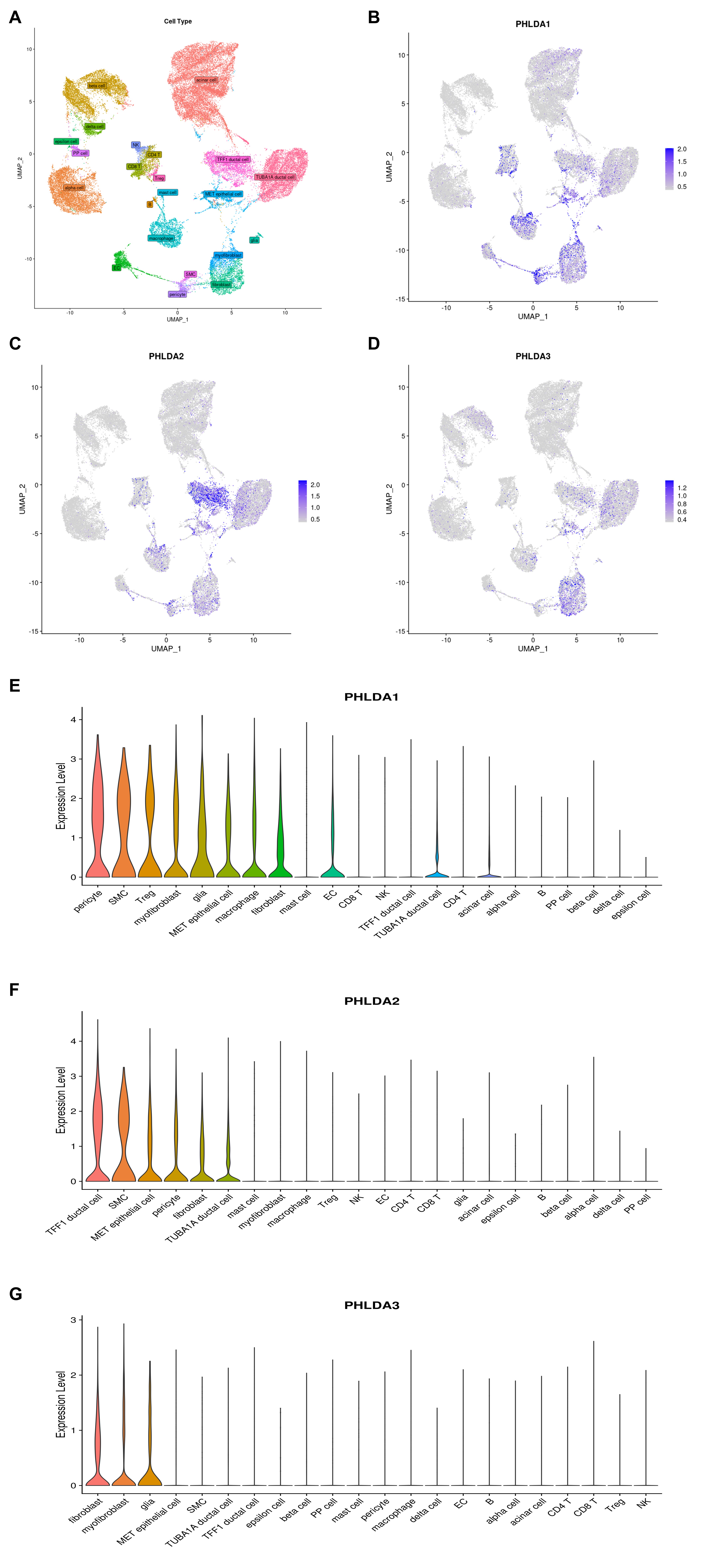

Supplement: Supplementary file 1 [file ijms-23-10316-s001.zip › Supplementary Figure S5 Single cell sequencing results of the differential expression of PHLDA family in pancreatic cancer tumor cells and immune infiltrating cells..jpg]

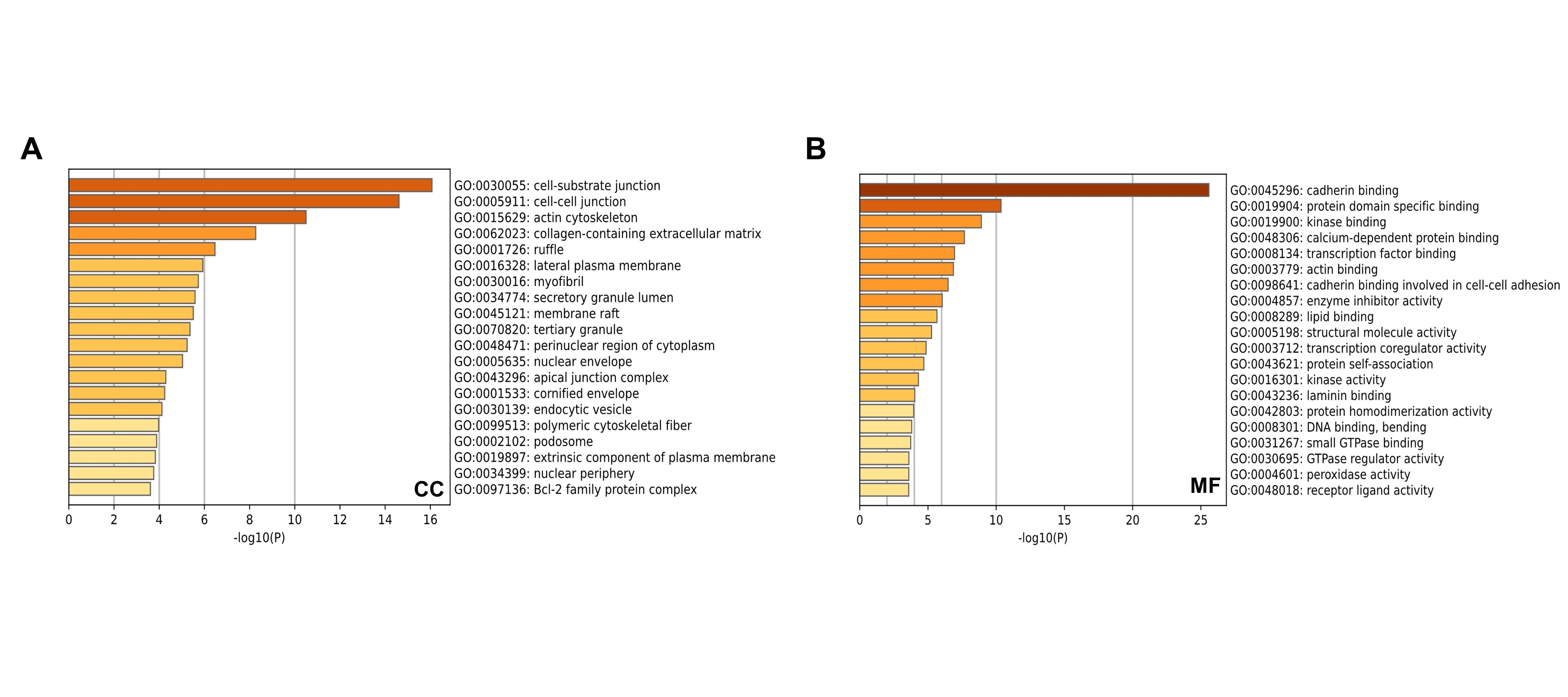

Supplement: Supplementary file 1 [file ijms-23-10316-s001.zip › Supplementary Figure S6 The GO enrichment of the CC terms and MF terms of the PHLDA family and its 600 co-expressed genes..jpg]
